# Supplementary material for: Disentangling top-down drivers of mortality underlying diel population dynamics of Prochlorococcus in the North Pacific Subtropical Gyre
Source: Nat Commun. 2024 Mar 7;15:2105. doi: 10.1038/s41467-024-46165-3 (PMC10920773; doi:10.1038/s41467-024-46165-3)
Supplement: Supplementary file 1 — Supplementary Information [file 41467_2024_46165_MOESM1_ESM.pdf]

Supplementary Information for

Disentangling top-down drivers of mortality underlying diel population dynamics of *Prochlorococcus* in the North Pacific Subtropical Gyre

Contents

Supplementary Note 1: Timeseries diel analysis **1**

Supplementary Note 2: Alternative mortality estimates **2**

Supplementary Note 3: ECLIP model and parameter inference **7**

Supplementary Note 4: Potential mechanisms to explain other losses of *Prochlorococcus* **22**

Supplementary Note 1: Timeseries diel analysis

We used the R package RAIN [1] (version 1.34.0) to evaluate whether the empirical *in situ* timeseries exhibited diel periodicity. RAIN uses non-parametric methods to evaluate periodicity and is optimized to focus on evenly sampled datasets with fewer than 100 measurements. In all cases we first detrended the timeseries. For *Prochlorococcus* cells, the size of data goes well above what is computationally feasible within RAIN, and sample times are not evenly spaced. Instead, we used the Lomb-Scargle Periodogram [2] implemented in R package *lomb* (version 2.1.0) to estimate the period (found to be between 0.977 and 1.023 days). Table S1 shows the results from the RAIN and Lomb-Scargle analyses. The timeseries of *Prochlorococcus* and infected *Prochlorococcus* was statistically significantly diel (at a level of $p<0.05$), the timeseries for T4- and T7-like viruses, and heterotrophic nanoflagellates was not statistically significantly diel (at a level of $p>0.05$).

Timeseries	Method	*p-value*	Significantly diel?
Prochlorococcus	Lomb-Scargle Periodogram	3.056296e-232	✓
Infected cells	RAIN	0.01042877	✓
% infected cells	RAIN	0.002237049	✓
Virus	RAIN	0.9064921	×
Grazer	RAIN	0.1904631	×

Table S1: Diel timeseries analysis. Diel timeseries analysis using Lomb-Scargle Periodogram for *Prochlorococcus* and RAIN for other timeseries, testing for the probability of whether timeseries are expected to exhibit periodicity of 1 day by chance.

Supplementary Note 2: Alternative mortality estimates

Modeling encounter rates and allometry

Viral encounter rate

Both *Prochlorococcus* and their viruses diffuse and the rates at which they may encounter each other can be estimated using biophysical models [3–5]. We assume the maximum encounter rate between diffusing spherical particles of two different sizes, r_{virus} and r_{cell} is given by the Smoulouski equation [6]. However, an encounter does not always lead to adsorption [3, 7] so we subject the maximum encounter rate to an efficiency of encounter term, ϵ_ϕ, as:

$$\phi(r_{virus}, r_{cell}) = \epsilon_\phi \left(4\pi \left(D_{cell} + D_{virus}\right) \left(r_{virus} + r_{cell}\right)\right) \tag{1}$$

where diffusion of each spherical particle of radius r is given by the classical relation [8]:

$$D(r_d) = \frac{K_b \cdot T}{6\pi \cdot \rho \cdot r_d} \tag{2}$$

with K_b, the Boltzmann constant, T is temperature (in Kelvin) and ρ is the dynamic viscosity of the medium. We assume $T = 25°\text{C}$ [9] and $\rho = 9.96 \times 10^{-10}$ kg μm^{-1} s^{-1} [3]. We assume that free viruses can adsorb to both susceptible and infected *Prochlorococcus* cells. Both cases result in losses of free virus particles. Susceptible cells that viruses adsorb to become infected. However, we assume that viruses that adsorb to infected cells do not alter the physiological responses of infected cells. As radius scales as $\approx$ [volume]$^{1/3}$, both diffusion and encounter rate are size-dependent [3].

Grazer encounter rate

We estimated encounter rates between grazers and prey by modeling the volume of water a swimming grazer will encounter as:

$$\psi = u\pi R^2 \tag{3}$$

where u is the swimming speed and R is the perception radius of the grazer. Here, the modeled phytoplankton, *Prochlorococcus* do not swim [10, 11]. We further account for differences in predator and prey size and encounter efficiency as:

$$\psi(r_{grazer}, r_{cell}) = \overbrace{\epsilon_\psi}^{encounter\ efficiency} \left(\overbrace{u(r_{grazer})}^{swimming\ speed} \; \pi(\overbrace{f_{detect} r_{grazer} + r_{cell}}^{sensing\ radius})^2 \right) \tag{4}$$

where we assume grazers swimming speed (μm d^{-1}) scales as a function of grazer equivalent spherical radius (μm) using the empirical relation derived by [12] as:

$$u(r_{grazer}) = 864 \times 10^{(6+u_1)} \left(\frac{2r_{grazer}}{10^4} \right)^{u_2} \tag{5}$$

with coefficients $u_1 = 0.39$ and $u_2 = 0.79$. We assume grazers detect prey within a detection sensing radius which is proportional to grazer radius with factor $f_{detect} = 3$ [3]. We also incorporate prey size into the perception radius [13].

Modeling carbon allometry

Ribalet et al. [14] correlate light-scattering to cell size and cell carbon quota using Mie theory. We define cellular elemental quotas (μg per cell) for carbon, C_S as:

$$C_S([\text{volume}]) = 220[\text{volume}] \cdot 10^{-9}. \tag{6}$$

We assume that both infected and susceptible cells have the same elemental quotas. That is $C_I = C_S$.

For the purposes of converting consumed biomass to grazer abundance we assume grazer quotas in μg per cell for carbon, based on the empirical relationship derived by Menden-Deuer and Lessard [15] for dinoflagellates as:

$$C_G(r_{grazer}) = 10^{-6} \cdot 0.76 \left(\frac{4}{3} \pi r_{grazer}^3 \right)^{0.819}. \tag{7}$$

Viral lysis estimates

iPolony estimates:

Estimates for viral-induced lysis based on infected cell data recorded on the cruise using the iPolony method are 0.35-4.8% [16], assuming 1-3 infection cycles occur during the turnover time for *Prochlorococcus* cells.

Viral encounter estimates

Naïve encounter rate estimates are made using the encounter rate theory outlined in equation 1 with baseline parameters assuming that total *Prochlorococcus* loss rates (ω) are between 0.3 and 1 per day. Note that this encounter rate estimation assumes instantaneous killing of *Prochlorococcus*. Assuming that the steady state abundance of T4- and T7-like cyanophages during the cruise was $V^* = 3.5 \times 10^8$ per L, that all cyanophage are infectious and all *Prochlorococcus* cells are susceptible to these cyanophage, and assuming variation in $\epsilon_\phi \in [10^{-5}, 10^0]$, $r_{virus} \in [20, 50]$ nm, $r_{cell} \in [0.17, 0.49]\mu$m, we find the proportion of *Prochlorococcus* losses via viral-induced lysis can be estimated as:

$$p_{vir} = \frac{\phi(r_{virus}, r_{cell})V^*}{\omega}. \tag{8}$$

Grazing estimates

Grazer encounter estimates

Naïve encounter rate estimates are made using the encounter rate theory outlined in equation 4 with baseline parameters assuming that total *Prochlorococcus* loss rates (ω) are between 0.3 and 1 per day. Note that this encounter rate estimation assumes instantaneous killing of *Prochlorococcus*. Assuming that the steady state abundance of heterotrophic nanoflagellates during the cruise was $G^* = 2.5 \times 10^5$ per L, and assuming parameter variation in $\epsilon_\psi \in [10^{-5}, 10^0]$, $f_{detect} \in [0, 6]$, $r_{grazer} \in [1, 10]\mu$m, $r_{cell} \in [0.17, 0.49]\mu$m, we estimate the proportion of *Prochlorococcus* losses via grazing as:

$$p_{graz} = \frac{\psi(r_{grazer}, r_{cell})G^*}{\omega}. \tag{9}$$

Carbon quota and predator growth rate estimate

Following [17] we use literature values to assume that heterotrophic nanoflagellate growth rate ξ is between 0.2 and 1.4 per day [18–20]. We can write an approximation for the growth rate of the heterotrophic nanoflagellate population, supposing that they only feed on *Prochlorococcus* cells, as:

$$\xi G = \epsilon \frac{C_S}{C_G} \psi (S + I) G \tag{10}$$

which can be rearranged to estimate the grazing clearance rate as:

$$\psi = \frac{\xi C_G}{\epsilon C_S (S + I)}. \tag{11}$$

We use a steady state approximation of $P^* = S^* + I^* = 1.75 \times 10^8$ *Prochlorococcus* cells per L and a steady state assumption of $G^* = 2.5 \times 10^5$ heterotrophic nanoflagellates per L during the cruise. Assuming variation in $\epsilon \in [0.2, 0.6]$, C_S and C_G we calculate the proportion of losses attributed to grazing as:

$$p_{graz} = \frac{\psi G^*}{\omega} \tag{12}$$

where ω represents total *Prochlorococcus* loss rates that we assume are between 0.3 and 1 per day.

FLB estimates

We used Fluorescently Labelled Bacteria (FLB) incubation experiments performed on the cruise [17] to estimate grazing mortality. These experiments measured the proportion of heterotrophic nanoflagellates containing FLB cells after an hour incubation. The proportion of heterotrophic nanoflagellates containing FLB showed diel variability and varied between 1-25% [17]. In order to use these measurements as the basis for estimating grazing mortality we propose that the population dynamics of FLB cells within incubation experiments can be modeled as:

$$\frac{dF}{dt} = -\alpha F G = -AF \tag{13}$$

where F is the abundance of FLB cells, α is the clearance rate of heterotrophic nanoflagellates feeding on FLB and A represents the grazing rate by heterotrophic nanoflagellates. Assuming that heterotrophic nanoflagellates graze on *Prochlorococcus* and FLB at the same rate, and assuming a constant heterotrophic nanoflagellate population, G, of 2×10^5 cells/L allows a grazing rate A to be computed as:

$$A = -\frac{1}{\tau} \log \left(\frac{F_E}{F_0} \right) \tag{14}$$

where τ is the experiment length (1 hour), the initial FLB abundance is $F_0 = 10^8$ cells per L, and the final FLB abundance is F_E. Assuming the observed proportion (0.01-0.25) of heterotrophic nanoflagellates containing FLB (Y) consume X FLB on average during the incubation period, we can calculate the final FLB abundance as:

$$F_E = F_0 - XYG. \tag{15}$$

We assume that X varies between 1 and 25, but, it is unclear exactly how many FLB were eaten per grazer during the 1 hour incubation. We assume that estimated grazing rates on FLB are equivalent

to grazing rates on *Prochlorococcus*. To convert to mortality, we assume that total *Prochlorococcus* loss rates, ω are between 0.3 and 1 per day and calculate proportional losses by grazing as:

$$p_{graz} = \frac{A}{\omega}. \tag{16}$$

Supplementary Note 3: ECLIP model and parameter inference

For completeness, and to help the reader, we repeat our descriptions of the ECLIP model in the supplementary text.

ECLIP: Ecological Community driven by Light, with Infection of Phytoplankton

The ECLIP model represents *Prochlorococcus* division and death where division is light-driven (where cell division is expected to occur at night [21, 22]) and death is controlled by viral lysis, grazing, and other density-dependent factors (Figure 1a). The *Prochlorococcus* population is structured by two states of infection: cells that are susceptible to viral infection (S) and cells that are infected (I) by viruses (V). Grazers (G) feed indiscriminately on both S and I classes. The dynamics of S, I, V and G numerical abundances over time are described by the following system:

$$\begin{aligned}
\frac{dS}{dt} &= \overbrace{\mu(t)S}^{division} - \overbrace{m_P S(S+I)}^{higher\text{-}order\ losses} - \overbrace{\phi SV}^{infection} - \overbrace{\psi SG}^{grazing} \\
\frac{dI}{dt} &= \overbrace{\phi SV}^{infected} - \overbrace{m_P I(S+I)}^{higher\text{-}order} - \overbrace{\eta I}^{viral\text{-}lysis} - \overbrace{\psi IG}^{grazing} \\
\frac{dV}{dt} &= \overbrace{\beta\eta I}^{viral\ production} - \overbrace{\phi(S+I)V}^{adsorption} - \overbrace{m_V V^2}^{higher\text{-}order\ losses} \\
\frac{dG}{dt} &= \overbrace{\epsilon\frac{N_P}{N_G}\psi(S+I)G}^{grazing\ on\ Prochlorococcus} - \overbrace{\gamma G}^{generalist\ grazing} - \overbrace{m_G G^2}^{higher\text{-}order\ losses},
\end{aligned} \tag{17}$$

where

$$\eta = \frac{24}{LP}, \text{with } LP \text{ is the latent period (in hours)} \tag{18}$$

$$\mu(t) = \mu_{ave}\,(\,1\ +\ \delta_\mu \sin(\,2\pi(t+\delta_t)\,)\,)\,. \tag{19}$$

Prochlorococcus have a diel-driven population division rate $\mu(t)$ whose proportional amplitude and phase are set by parameters δ_μ and δ_t, and $t = 0$ represents 06:00:00 local time (see Figure S1). *Prochlorococcus* have a nonlinear loss rate, m_P, dependent on total phytoplankton population size to implicitly represent niche competition [23]. Viruses infect susceptible *Prochlorococcus* at a rate ϕ and create β new virions that are released into the environment upon cellular lysis, which occurs after an infection latent period of $\frac{1}{\eta}$. Grazing upon *Prochlorococcus* is non-preferential with respect to infection status and occurs at a rate ψ with a Gross Growth Efficiency (GGE) ϵ proportional to the fraction of nitrogen contents in a *Prochlorococcus* cell ($N_P = 5.01 \times 10^{-9}\mu$g N cell^{-1}) and a grazer ($N_G = 6.53 \times 10^{-6}\mu$g N cell^{-1}). We introduce γ as a parameter to denote whether grazers act as specialists ($\gamma = 0$) or generalists ($\gamma > 0$), where the term represents net additional gains to the grazer from non-*Prochlorococcus* prey sources

after accounting for respiratory costs. Generalist strategies may include ingesting other phytoplankton, heterotrophic bacteria, or other grazers through intraguild predation. Grazer and viral losses are both characterized by a nonlinear loss term to avoid structurally biasing the model to favour one of these types of *Prochlorococcus* predators [24] and to avoid competitive exclusion. A full list of parameters are shown in Table S2.

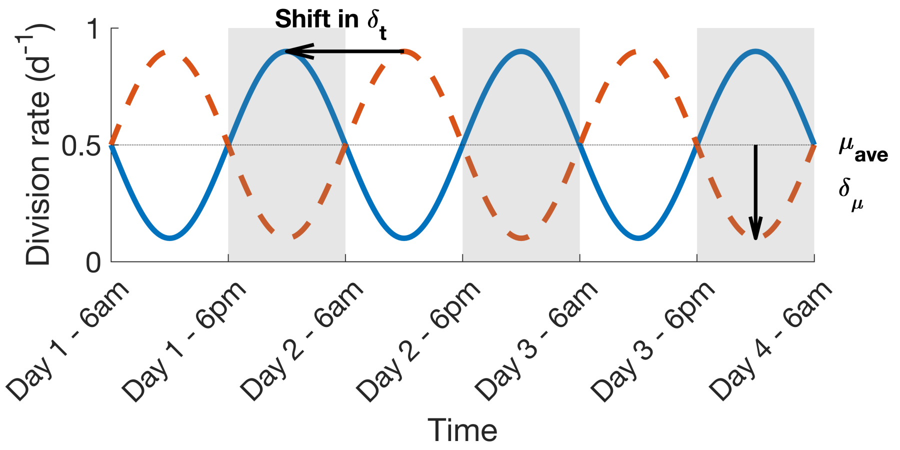

Figure S1: Diel-driven division rate. Solid blue and dashed orange lines are example division rate curves to graphically explain how the different parameters used in the diel-driven division rate function in ECLIP alter the model division rate. The parameters for division rate are represented by the average division rate over 1 day, μ_{ave}, the amplitude of the sinusoidal oscillations, δ_μ, and the phase of the sinusoidal signal, δ_t. The two curves differ in their value of δ_t; the orange curve has $\delta_t = 0$, while $\delta_t = 0.5$ days for the blue curve.

Defining the specialism-generalism gradient

We define the degree of generalism ($d_{\text{generalism}}$) as the ratio of growth performed by grazers derived from other sources relative to that derived from both other sources and from consuming *Prochlorococcus*:

$$d_{\text{generalism}} = \frac{\gamma}{\epsilon \frac{N_P}{N_G} \psi(S+I) + \gamma}. \tag{20}$$

Model inferred mortality

Total *Prochlorococcus* loss rates are defined as:

$$m_{total} = m_{\text{lysis}} + m_{\text{grazing}} + m_{\text{other}} \tag{21}$$

where:

$$\begin{aligned} m_{\text{lysis}} &= \eta I \\ m_{\text{grazing}} &= \psi(S+I)G \\ m_{\text{other}} &= m_P(S+I)^2. \end{aligned} \tag{22}$$

Event		Variable	Units
S and I	Average division rate	μ_{ave}	d^{-1}
	Amplitude division rate	δ_μ	unitless
	Phase of the division rate	δ_t	d
	Higher order loss rate	m_P	$L(\text{cell} \cdot d)^{-1}$
V	Adsorption	ϕ	$L\ (\text{virus} \cdot d)^{-1}$
	Latent period	LP	hours
	Burst size	β	viruses cell^{-1}
	Higher order viral losses	m_V	$L(\text{virus} \cdot d)^{-1}$
G	Clearance rate	ψ	$L(\text{grazer} \cdot d)^{-1}$
	Degree of generalism	γ	d^{-1}
	Higher order grazer losses	m_G	$L(\text{grazer} \cdot d)^{-1}$

Table S2: Parameters of the the ECLIP model

We defined the proportion of each mortality processes as follows:

$$\begin{aligned} r_{\text{lysis}} &= \text{mean}\left(\frac{m_{lysis}}{m_{total}}\right) \\ r_{\text{grazing}} &= \text{mean}\left(\frac{m_{grazing}}{m_{total}}\right) \\ r_{\text{other}} &= \text{mean}\left(\frac{m_{other}}{m_{total}}\right). \end{aligned} \tag{23}$$

Diel adsorption rates

We incorporated a diel adsorption rate into our analyses in Figure 9 to see if this would better represent the observed population dynamics of infected *Prochlorococcus* cells. We did this by modulating the average inferred adsorption rate with a time dependent step function such that adsorption rates are highest at dusk, and lowest at dawn - consistent with lab experiments and associated modeling between cyanophage and *Prochlorococcus* [25, 26].

$$\phi(t) = \begin{cases} 0.5\phi & 12 \text{ a.m.} \leq t \leq 12 \text{ p.m.} \\ 1.5\phi & 12 \text{ p.m.} \leq t \leq 12 \text{ a.m.} \end{cases} \tag{24}$$

Model-data integration

General parameter inference implementation

We determined the parameter sets for the ECLIP model that optimized the fit of the model dynamics to field measurements by using Markov Chain Monte Carlo (MCMC) implemented in the probabilistic inference package Turing [27] in the Julia language [28]. MCMC is a class of Bayesian inference algorithms that aims to infer the probability distribution of the model parameters given the model equations,

environmental data and prior beliefs [29]. We used the No-U-Turn Sampler (NUTS) implemented in Turing to sample the posterior distributions [30]. Classic Hamiltonian Monte Carlo (HMC) algorithms are sensitive to the number of steps of the simulation reducing the performance of the algorithm in high-dimension problems. NUTS is a performant alternative algorithm for MCMC that eliminates the need to set the number of steps, allowing better performance for high-dimensional parameter space and non-linear dynamical models [30].

We implemented the parameter estimation in two steps. First, we estimated the three parameter posterior distributions (μ_{ave}, δ_μ and δ_t) for the division rate function (see Supplementary Information equation 19) using a model-inferred *Prochlorococcus* division rate estimated for the same oceanographic campaign by [31]. In a second step, we used the division parameter posteriors as priors for another parameter inference to estimate the whole set of model parameters (μ_{ave}, δ_μ, δ_t, m_P, m_V, m_G, ϕ, β, LP, and ψ) and the abundance measurements for total and infected *Prochlorococcus* cells, cyanophages and heterotrophic grazers. We repeated this second step six times by fixing the generalism term at $\gamma = 0$, 0.01, 0.05, 0.1, 0.2 and 0.5 respectively, to account to different levels of non-*Prochlorococcus* contributions to grazing.

For each implementation, we ran 4 MCMC chains for 4000 iterations with a 2000 iteration warm-up period (total iterations = 6000) and a target acceptance ratio of 0.65, allowing the algorithm to reach a stationary state. For the model $\gamma = 0.5$, we obtained chains with high autocorrelation. To decrease the autocorrelation we ran 16 independent chains of 4000 iterations and combined them in four longer chains of 16000 iterations. We then thinned these longer chains [32] by sampling every 4 iterations to obtain 4 MCMC chains of 4000 iterations with lower autocorrelation.

Chain convergence analysis

To assess the convergence of the MCMC chains, we computed different indicators for each implementation [33]. For each parameter, the inter-variability of the chains was computed as the $\widehat{R}$. When the set of chains converged to the same posterior distribution, $\widehat{R}$ should be centered around 1 (with upper bound of $\widehat{R} = 1.1$), suggesting good convergence (Figure S5). We computed the ratio of effective sample size (N_{eff}) to the total iteration size ($N = 4000$) to search for potential sampling issues. Low ratio $\left(\frac{N_{eff}}{N} < 0.1\right)$, indicates potential sampling problems with higher chain auto-correlation, suggesting divergence (Figure S5). Additionally, we also computed auto-correlation for each parameter chains of the ECLIP model (Figure S8).

Division function parameter inference

We fitted our division function (Supplementary Information equation 19) to the timeseries of division rates estimated by integrating *in situ* flow cytometry measurements with the pmb size-structured *Prochlorococ-*

cus model described in [31]. Daily division rates were estimated using a size-structured matrix population model that mechanistically describes changes in microbial cell size distribution over the day-night cycle [31]. In brief, the model assumes that changes of the cell size distribution is driven by three interconnected biological processes: carbon fixation via photosynthesis, carbon loss via respiration and exudation and cell division. We select the pmb model with a power-law size dependence on carbon fixation since there is strong evidence of an allometric growth rate in natural populations of *Prochlorococcus* [34]. The model was fit to a logarithmically spaced discrete cell size distribution of *Prochlorococcus* provided by SeaFlow, using the same priors of model parameters used in [31].

This implementation helped us to refine the division priors for the whole model parameter estimation step. We used wide uniform priors for the three parameters of the division function: μ_{ave}, δ_μ and δ_t (Figure S2, Table S3). The 4 chains converged ($\widehat{R} \approx 1$, with no auto-correlation), to narrower distributions centered around $\mu_{ave} = 0.48\ d^{-1}$, $\delta_\mu = 0.75\ d^{-1}$ and $\delta_t = 0.83\ d$. Our division function predicted well the division rates from [31] (Figure S3).

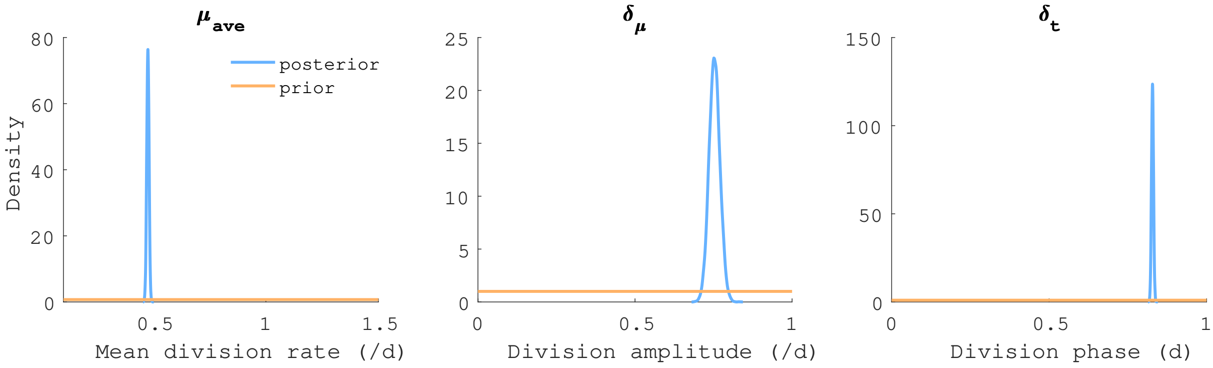

Figure S2: Priors and posteriors distribution for the division function parameters. We used wide uniform priors (orange) allowing to search a wide space for each parameter. Posteriors (blue) had narrower distributions that helped us to reduce the parameter range for the whole parameter inference step.

ECLIP parameters inference implementation

To estimate the whole parameter set for the ECLIP model, we used environmental abundance data from the SCOPE HOE-Legacy 2A cruise (see Empirical data section, 2.3 in the main text, for more information). Specifically, we used total and infected *Prochlorococcus* cells, free cyanophage and heterotrophic bacteria. We inferred parameters of the ECLIP model for six levels of generalism: $\gamma = 0$, 0.01, 0.05, 0.1, 0.2, and 0.5 day^{-1}. For each implementation, we used the same priors for model parameters and log-likelihood (see next section for more information on the priors). Comparison between posteriors of the 6 models and the priors is shown in Figure S4.

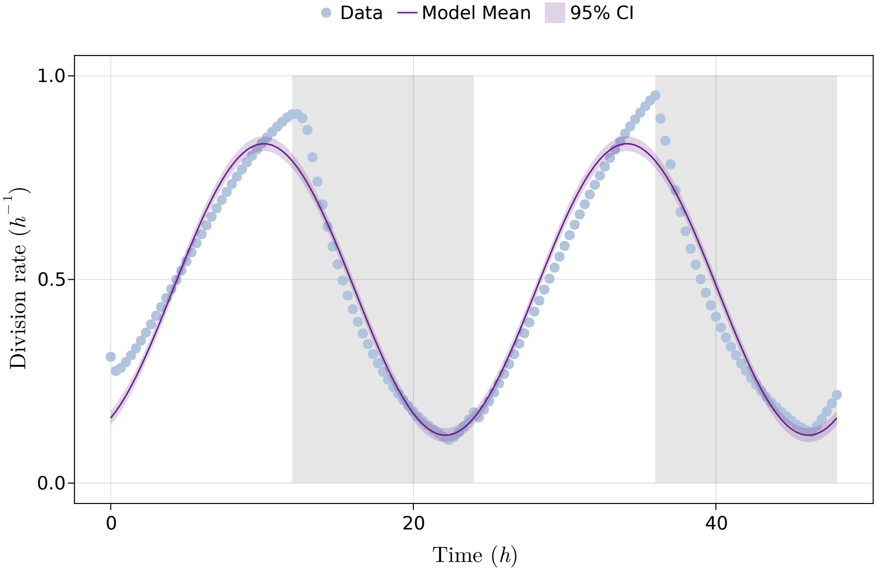

Figure S3: Division function fits. Our periodic function (purple) well represented the estimated division rate (blue dot) from [31]. Gray vertical shaded areas represent the night periods.

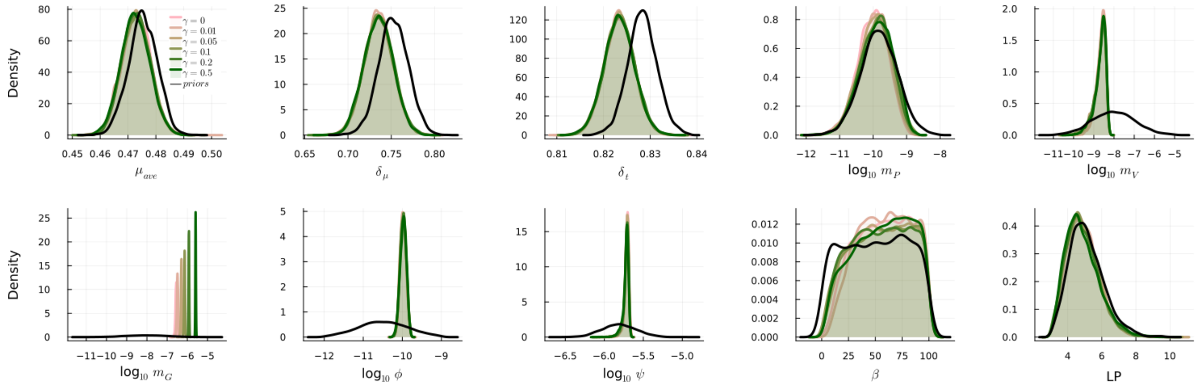

Figure S4: Comparison of parameter prior and posterior estimates in ECLIP. Prior distributions are represented in solid black lines, while posteriors are represented in colored solid lines (from pink $\gamma = 0$ d^{-1} to dark green $\gamma = 0.5$ d^{-1}).

Priors and parameter ranges

We defined priors for both inference implementations, for each of the model parameters and the standard deviation σ of the likelihoods (Table S3). The ECLIP model is prone to identifiability problems due to parameter correlations, possibly leading to performance and convergence issue of the MCMC algorithm. Therefore, we weighted higher probability space for some parameters by setting Normal or log-Normal priors instead of Uniform priors for the majority of the parameters, while setting wide variance for each prior, to explore the larger parameter space as possible given ecological realistic ranges. Additionally, for the whole implementation, we fixed three parameters to reduce parameter identifiability issues: the gross growth efficiency ($\epsilon = 0.3$, [35]), and the fraction of Nitrogen content for *Prochlorococcus* ($N_P = 5.01 \times 10^{-9} \mu$g N cell^{-1}) and grazers ($N_G = 6.53 \times 10^{-6} \mu$g N cell^{-1}).

Implementation	Variable	Bounds	Priors
Division function	μ_{ave}	0.1 – 1.5	Uniform
	δ_μ	0 – 1	Uniform
	δ_t	0 – 1	Uniform
	$\sigma_{likelihood,div.}$	$\mathbb{R}^+$	InverseGamma(3,2)
Whole model	μ_{ave}	0.45 – 0.6	Normal$(0.475, 0.005)$
	δ_μ	0.3 – 1	Normal$(0.753, 0.017)$
	δ_t	0.2 – 1	Normal$(0.828, 0.003)$
	m_P	10^{-12} – 10^{-8}	LogNormal$\left(log\left(1.56 \times 10^{-10}\right), 1.249\right)$
	ϕ	10^{-12} – 10^{-9}	LogNormal$\left(log\left(3.2 \times 10^{-11}\right), 1.48\right)$
	LP	3 – 12	LogNormal$(log\,(5)\,, 0.2)$
	β	0 – 100	Uniform
	m_V	10^{-11} – 10^{-5}	LogNormal$\left(log\left(1.01 \times 10^{-8}\right), 2.45\right)$
	ψ	10^{-10} – 10^{-4}	LogNormal$\left(log\left(1.55 \times 10^{-6}\right), 0.5\right)$
	m_G	10^{-11} – 10^{-5}	LogNormal$\left(log\left(1 \times 10^{-6}\right), 0.776\right)$
	$\sigma_{loglikelihood,P}$	$\mathbb{R}^+$	InverseGamma(6,1)
	$\sigma_{loglikelihood,I}$	$\mathbb{R}^+$	InverseGamma(6,1)
	$\sigma_{loglikelihood,V}$	$\mathbb{R}^+$	InverseGamma(6,1)
	$\sigma_{loglikelihood,G}$	$\mathbb{R}^+$	InverseGamma(6,1)

Table S3: ECLIP Priors and parameter ranges. Parameters and priors explored for parameter inferences.

Adding diel-dependent adsorption rates

In Figure S11 we added step-wise diel-dependent adsorption rates to the core ECLIP models, as described earlier in this Supplementary Note and in Supplementary Information equation 24.

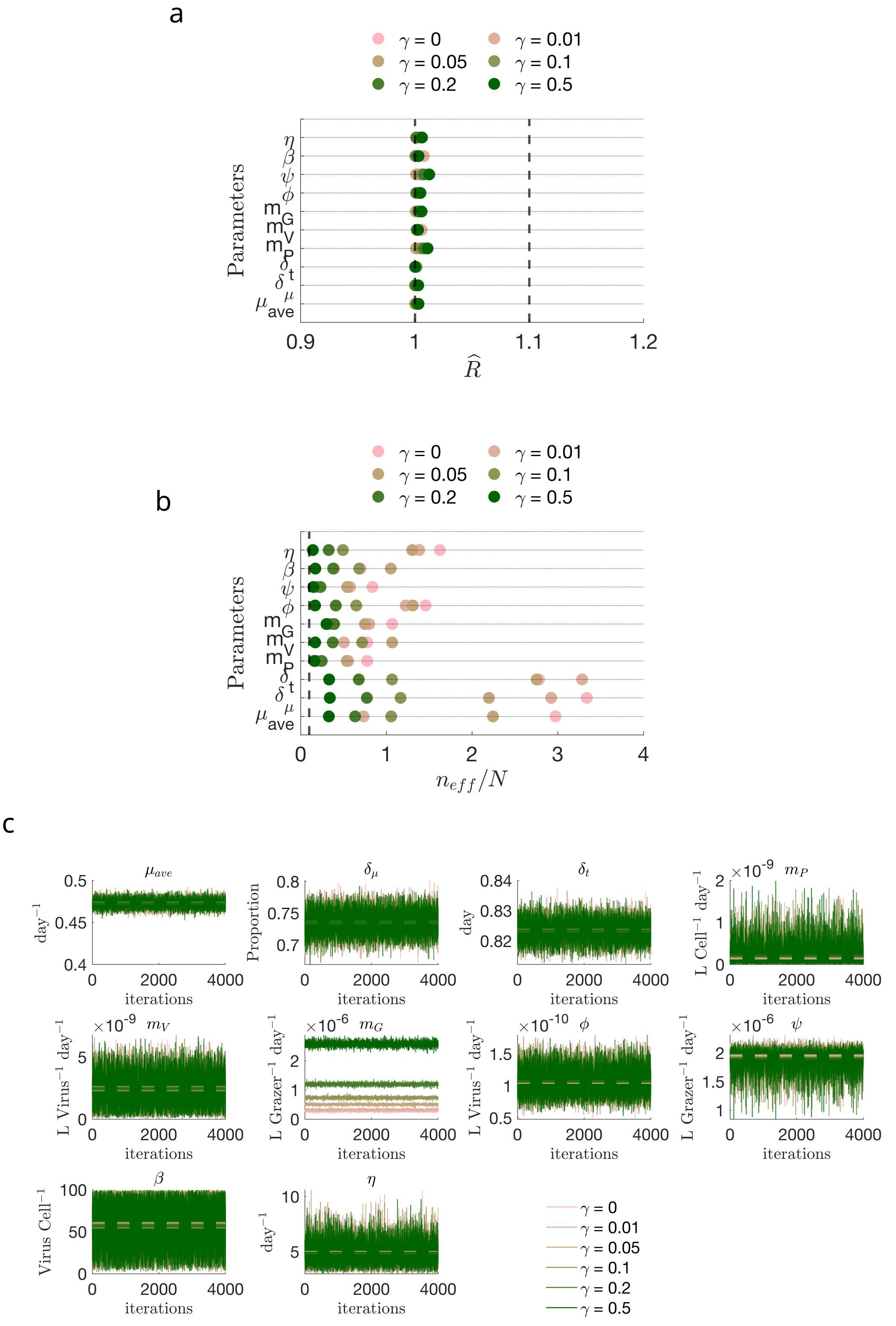

Figure S5: Assessing MCMC convergence and efficiency for ECLIP models. (a) $\widehat{R}$ convergence diagnostics. (b) Ratio of effective sample size to sample size. (c) Visualizing MCMC parameter chains for each of the ECLIP models (note density plots from chains are shown in Figure 5c). Full details of parameter bounds are shown in Table S2.

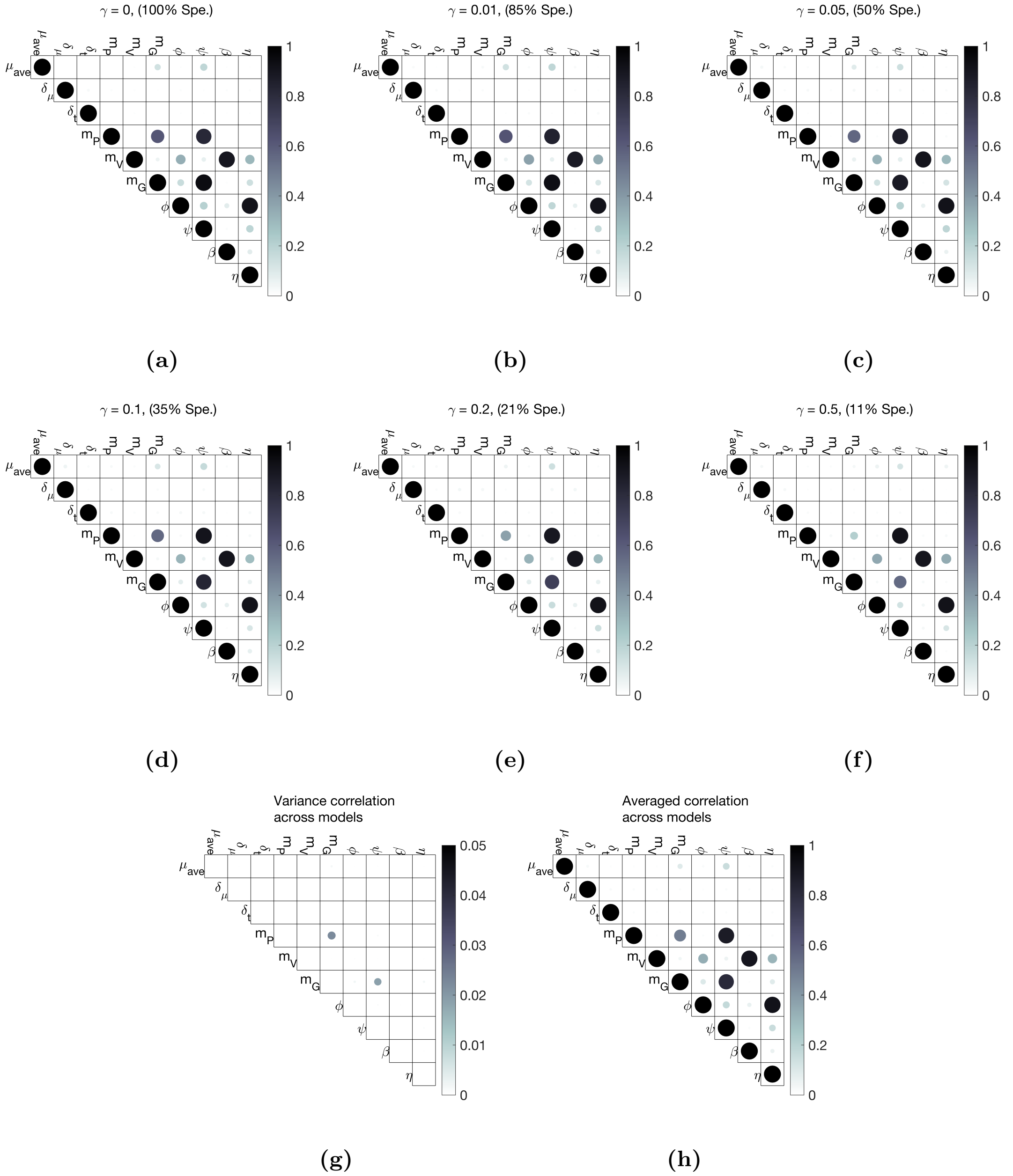

Figure S6: Identified parameter correlation in ECLIP models. Pearson correlation between parameters in MCMC chains for models with: (a) $\gamma = 0$ d^{-1}, (b) $\gamma = 0.01$ d^{-1}, (c) $\gamma = 0.05$ d^{-1}, (d) $\gamma = 0.1$ d^{-1}, (e) $\gamma = 0.2$ d^{-1}, (f) $\gamma = 0.5$ d^{-1}. (g) Pearson correlation between parameters across all MCMC chains together. (h) Average of the assessed correlations between parameters across grazer strategy models (a-f).

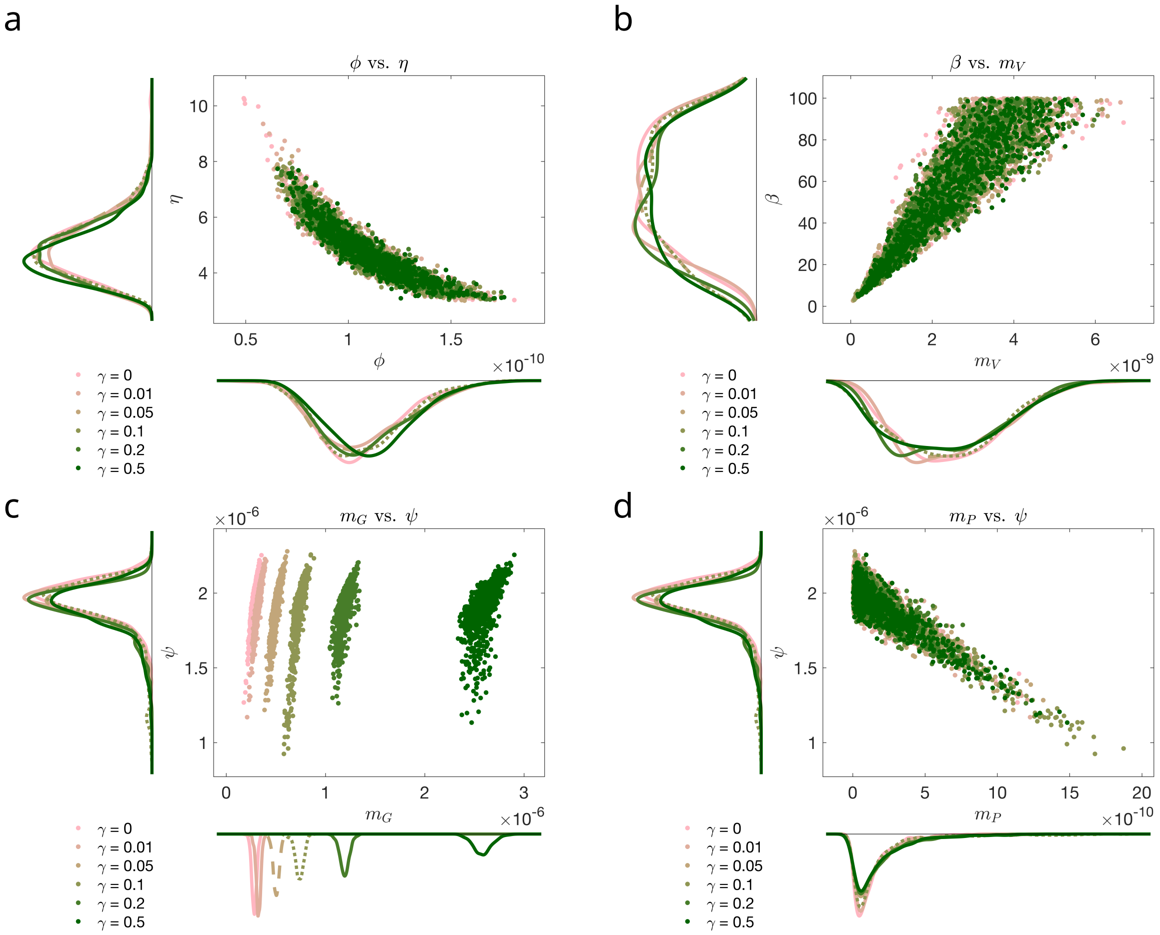

Figure S7: Examples of parameter covariance in ECLIP. Scatterplots of highly correlated ecological parameters: (a) adsorption rate (ϕ) vs lysis rate (η), (b) burst size (β) vs. viral higher order loss (m_V), (c) grazer higher order loss (m_G) vs. grazing rate (ψ), (d) *Prochlorococcus* higher order loss (m_P) vs. grazing rate (ψ).

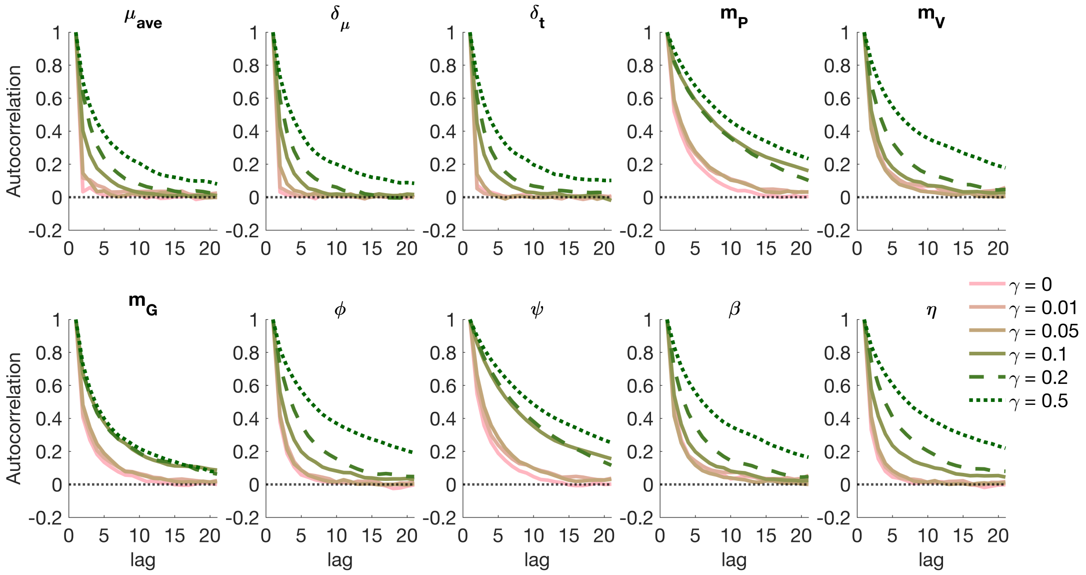

Figure S8: Parameter autocorrelation across ECLIP models. Autocorrelation was calculated as the cross-correlation of the chain with itself. Strong autocorrelation is leading to patterns or periodicity in the chain and suggest bad convergence. Our 6 models have relatively low autocorrelation for each parameter suggesting convergence.

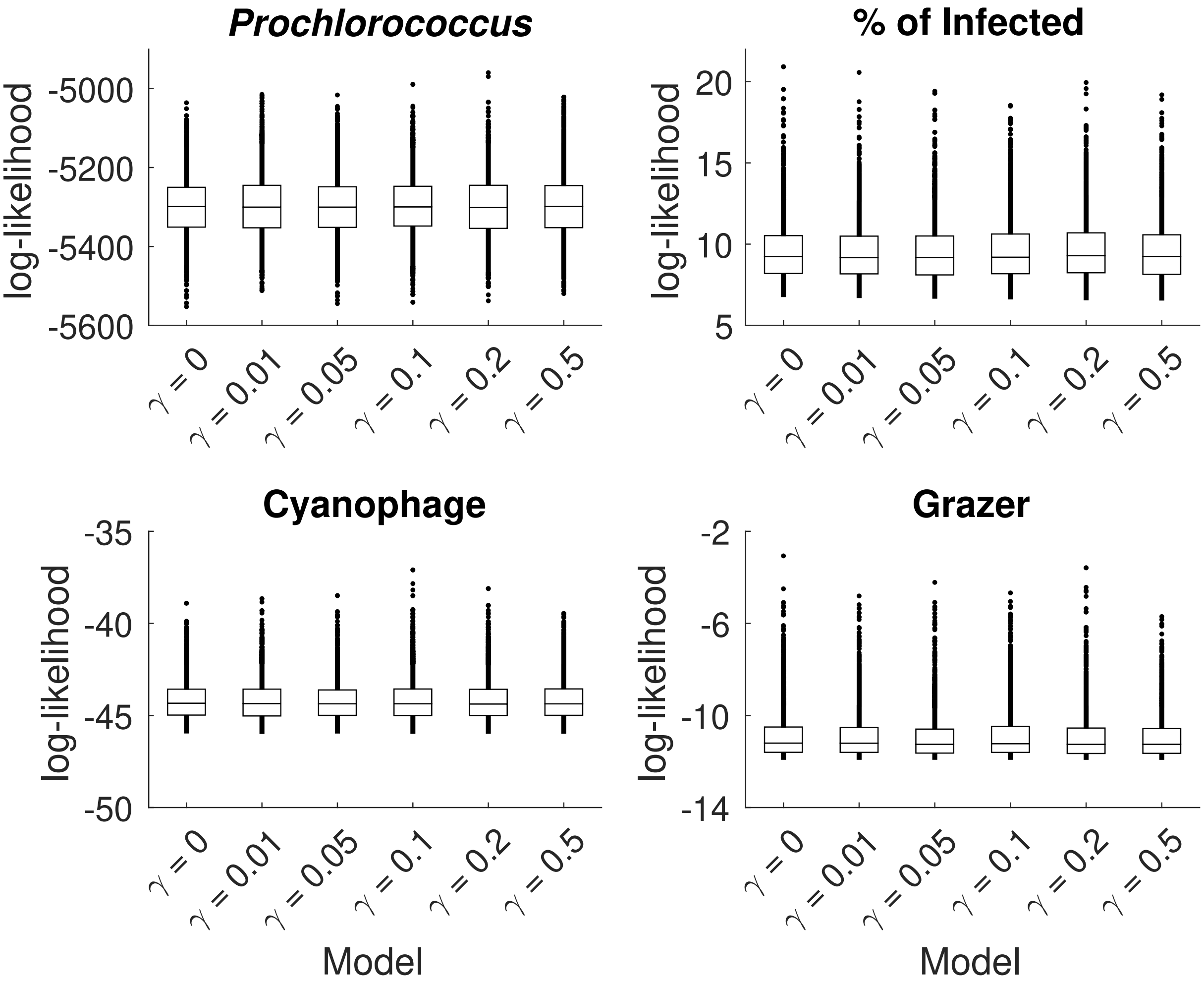

Figure S9: Negative log-likelihood for *Prochlorococcus*, % of infected *Prochlorococcus*, cyanophage and grazer across the 6 models. The negative log-likelihood function was calculated, for each chain step, as $LL(x_i) = \sum_i^n -log(p(x_i))$, where $p(x_i)$ is the model probability density function following a log-normal distribution and n the number of observations. The best model has the minimum LL. LL can only be compared between models for the same observations channels (*Prochlorococcus*, % of infected, Cyanophage or Grazer) and not between channels. The 6 models have the same ability to reproduce the observations for each chanels. Boxplots show the variability of the LL across the MCMC chain.

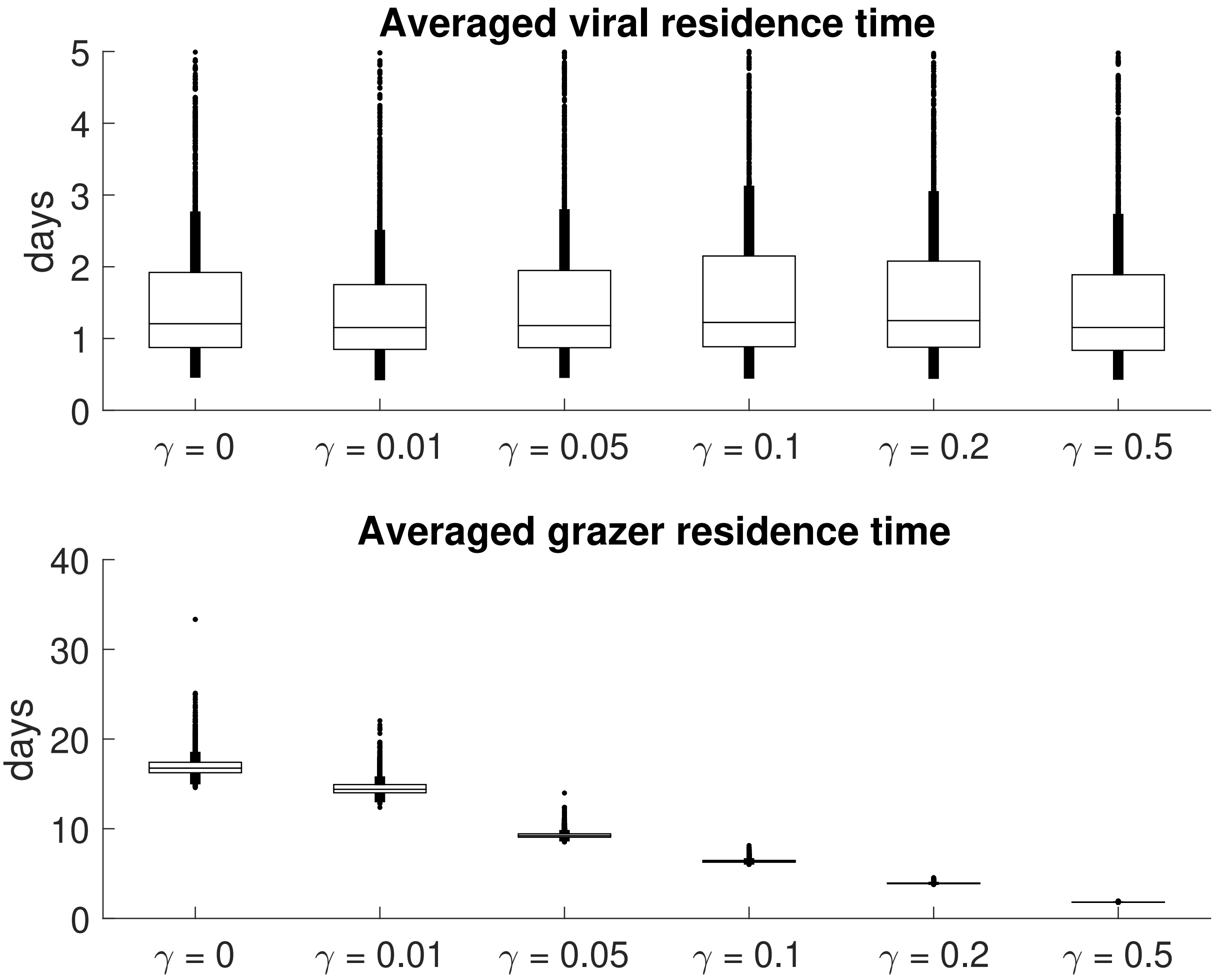

Figure S10: Averaged residence time of cyanophage (upper panel) and grazer (lower panel). Viral and grazer residence times were calculated, for each chain step, as $T_V = mean(1/(m_V \cdot V(t))$ and $T_G = mean(1/(m_G \cdot G(t))$ respectively. Median virus residence times are similar across the 6 models and range from 1.16 to 1.22 days, whereas median grazer residence times decreased from model 16.8 days (model $\gamma = 0$) to 1.8 days (model $\gamma = 0.5$). Boxplots show the variability of the log-likelihood across the MCMC chain.

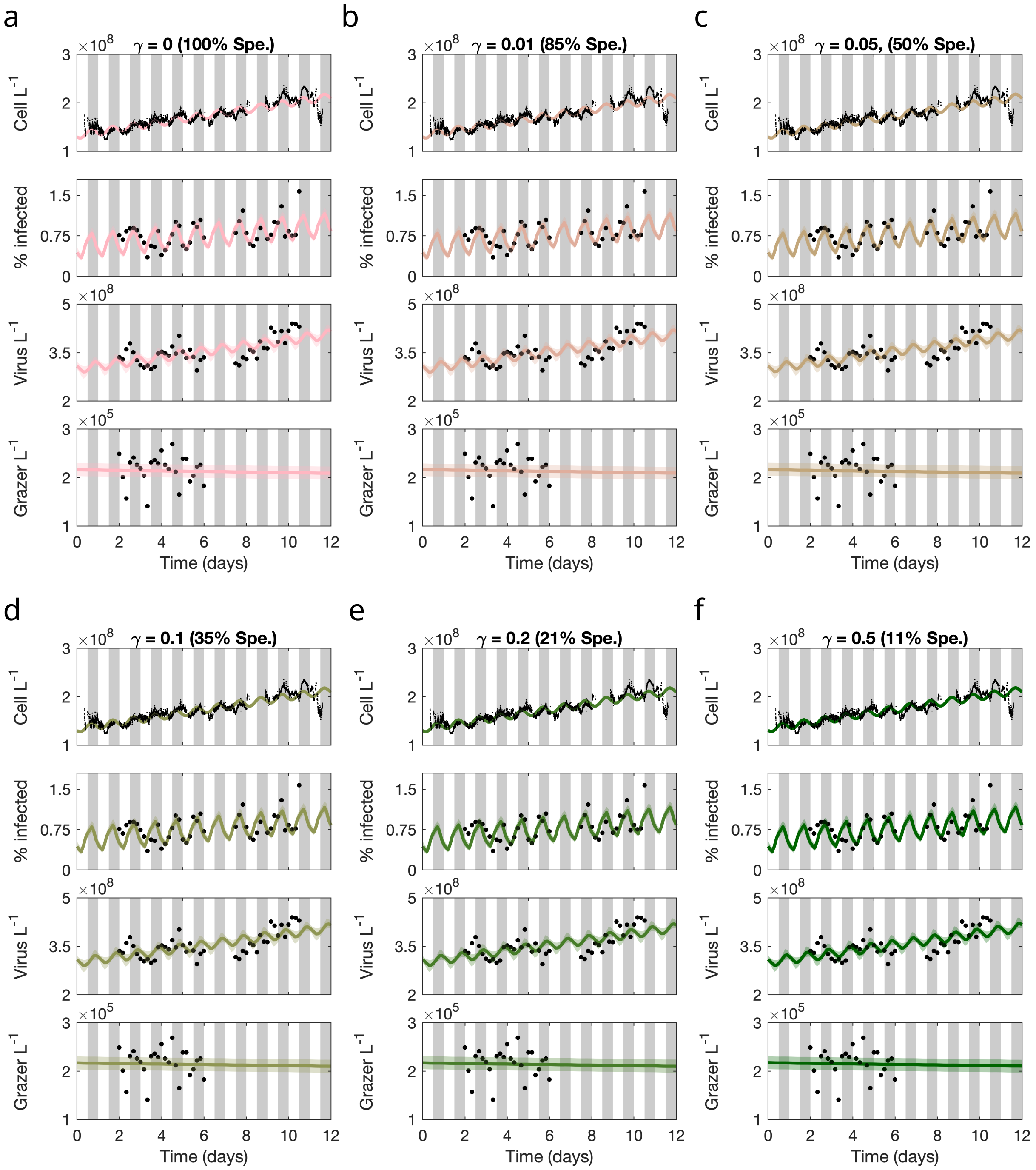

Figure S11: Models with diel adsorption rates across the specialism-generalism gradient fit empirical data. ECLIP models are compared against empirical data in black. Model lines represent the median MCMC solution within 95% CI range found by the converged chains, shown as bands with colours representing the choice of γ. Data signals include *Prochlorococcus* cell abundances (top), the percentage of infected *Prochlorococcus* cells, the abundance of free viruses and the abundance of heterotrophic nanoflagellate grazers (bottom). The models were fitted against detrended data; for visualization we have added these trends to the model solutions. Grey bars indicate nighttime. Model solutions with: (a) $\gamma = 0$ (grazers act as specialists), (b) $\gamma = 0.01$, (c) $\gamma = 0.05$, (d) $\gamma = 0.1$, (e) $\gamma = 0.2$, (f) $\gamma = 0.5$ day^{-1}. The degree of grazer specialism (Spe.) is shown in parentheses above each subplot.

Cells killed per L per day

$\gamma = 0$	Sensitivity ratio	$\gamma = 0.5$	Sensitivity ratio
μ_{ave}	0.352398	μ_{ave}	1.4308
ψ	0.30848	m_G	1.14668
m_G	0.155663	ψ	0.879708
ϕ	0.0253106	ϕ	0.106688
m_V	0.0131785	m_V	0.0553374
β	0.0129329	β	0.0548546
m_P	0.00773625	m_P	0.0498879
η	0.000203441	η	0.00402116
δ_μ	2.44229e-5	δ_μ	4.18003e-5
δ_t	7.90161e-11	δ_t	8.03853e-11

Lysis:grazing losses

$\gamma = 0$	Sensitivity ratio	$\gamma = 0.5$	Sensitivity ratio
ϕ	0.352165	m_G	1.2543
ψ	0.352145	μ_{ave}	1.14596
β	0.179552	ψ	1.00171
m_V	0.176093	ϕ	0.257941
m_G	0.176073	β	0.131193
η	0.0319323	m_V	0.127178
μ_{ave}	0.0308442	m_P	0.0449321
δ_μ	5.37848e-5	η	0.0277362
m_P	2.70933e-7	δ_μ	4.6763e-5
δ_t	1.76728e-11	δ_t	2.70028e-11

Table S4: Assessing parameter sensitivity to model mortality outcomes. We assess parameter sensitivity as applied to the parameter sets inferred via MCMC from the specialist grazing model ($\gamma = 0$) and the model in which grazers act most as generalists ($\gamma = 0.5$). Parameters are ordered from most to least sensitive for each evaluation, where sensitivity ratios are defined for outcomes Y (either cells killed per L per day, or the lysis:grazing ratio) and each MCMC-inferred parameter X, based on increasing and decreasing X by 20% as: $J = |\log_{10}\left(\frac{Y(1.2X)}{Y(0.8X)}\right)|$. This function denotes symmetric differences in magnitude (x10) between the values $Y(1.2X)$ and $Y(0.8X)$, such that $J = 1$ means that the outcome $Y(1.2X)$ is ten times larger than $Y(0.8X)$, or that $Y(0.8X)$ is ten times larger than $Y(1.2X)$; while $J = 0$ would suggest $Y(0.8X) = Y(1.2X)$. We varied one parameter at a time, while keeping all other parameters at their inferred values. Parameters are defined in Table S2.

Model outcomes sensitivity analysis

To assess the robustness of our findings regarding *Prochlorococcus* mortality we performed sensitivity analyses. To do this we focus on two model versions across the grazer specialism-generalism gradient, that with $\gamma = 0$ and that with $\gamma = 0.5$. We took the MCMC inferred life-history parameter sets from each of these models and ran simulations lasting 1,000 days (to avoid transient dynamics), and used dynamics on the final day to evaluate baseline levels of mortality (cells killed per L per day) and mortality source (lysis:grazing loss ratio). To assess the sensitivity of the ecological life-history traits in our model we chose to vary one parameter at a time by increasing or decreasing it by 20% and comparing changes in cells killed per L per day and lysis:grazing ratios to compute a sensitivity ratio: $J = |\log_{10}\left(\frac{Y(1.2X)}{Y(0.8X)}\right)|$.

This ratio assesses the magnitudinal differences in measured outcomes (Y) between when each parameter (X) is increased by 20% and decreased by 20%. Rank-ordered sensitivity ratio values are shown in Table S4. We observe that some parameters are more sensitive than others; and that mortality outcomes in the more generalist model ($\gamma = 0.5$) are overall more sensitive than in the specialist model ($\gamma = 0$).

Supplementary Note 4: Potential mechanisms to explain other losses of *Prochlorococcus*

Undercharacterized ecological interactions

Mixotrophic nanoflagellates contribute to *Prochlorococcus* grazing, with measurements suggesting smaller contribution relative to the heterotrophic nanoflagellates [17, 36] – but, still likely significant [37, 38]. Evaluating *in situ* mixotrophy is an ongoing challenge. Unknown viruses not quantified by the iPolony method (e.g., cyanophage without an identifiable DNA polymerase gene - see [39]), and other grazer types including larger consumers may contribute to 'other mortality'. Additionally, in ECLIP viral-induced and grazing-induced losses utilise a contact-driven model, analogous to 'Type I' functional responses. Mechanistic changes in functional responses and/or responses to light may drive distinct interaction rates (and aggregate mortality) even given the same set of viruses and grazers.

Aggregation and sinking of picoplankton

Picoplankton (and their viruses) are implicated as important contributors to export in large scale analyses e.g., [40, 41], though Guidi et al. [41] suggest *Synechococcus* rather than *Prochlorococcus* abundances appear correlated to export. Less is known about microscale processes leading to picoplankton export contributions – *Prochlorococcus* cells can be sustained in laboratory culture for month-long experiments, suggesting limited cell sedimentation. Generally, conceptual models of ocean ecology don't include sinking out of picoplanktonic populations – assuming it is inconsequential [42]. However, particle aggregation could be stimulated via sloppy feeding or viral lysate [24, 43]. As viral lysate from picocyanobacteria is labile [44–46] aggregation and sinking could be stimulated indirectly via heterotrophic bacterial growth. Particle attachment and aggregation could also be stimulated by TEP (Transparent exopolymer particles), produced in xenic *Prochlorococcus* cultures [47, 48]. TEP production can be stimulated by high light intensity, characteristic of the surface ocean, and its production appears linked to loss rates [47]. Picoplankton export could also be attributed to mineral ballasting [42] – both *Synechococcus* [49, 50] and *Prochlorococcus* [50, 51] have been suggested to accumulate biogenic silica.

Physiological stress(es)

Other cyanobacterial loss mechanisms include physiological stress, induced for example, by high-level irradiance associated with ultraviolet radiation [52, 53] and refraction of light through surface waves that could lead to photodamage via the flashing effect [54, 55]. Other abiotic factors including nutrient deficiency [56], metal toxicity [57–59], and thermal variations [60, 61] can also contribute to increased stress, though we do not expect significant thermal variations in the NPSG (mean seasonal changes are +/- 3°C (23.5-26.5 °C)). Stressors often results in the generation of reactive oxygen species, that cause oxidative stress and can cause DNA damage. Reactive oxygen species are also used as a signalling pathway for programmed cell death in photosynthetic microbes [62–64], though evidence is lacking for a programmed cell death pathway in *Prochlorococcus* and sympatric heterotrophic bacteria are thought to alleviate this stress [65, 66]. We note evidence is also lacking for toxin-antitoxin systems, common across bacteria and archaea which could lead to cell loss, in *Prochlorococcus* [67].

Population heterogeneity

Microbes experience senescence and aging, leading to intracellular accumulation of damage through their life cycle [68], which may lead to asymmetric division [69]. Unlike other microorganisms, *Prochlorococcus* has no resting stages and relies on heterotrophic bacteria to survive nutrient starvation in cultures [70], though the heterotrophic bacteria they form associations with *in situ* differ from those found in culture which tend to be copiotrophic [71]. *Prochlorococcus* populations are combinations of ecotypes which respond differently to environmental stressors [72–77]. Heterogeneity within the *Prochlorococcus* population, potentially via vertical displacement, may mean there are differing mortality responses at the individual or strain-level vs. at the scale of total population. Additionally, competition between ecotypes and with other phytoplankton in the same niche (like *Synechococcus* or picoeukaryotes) may also increase stresses, especially in a nutrient limited oligotrophic environment like the NPSG.

References

1. Thaben, P. F. & Westermark, P. O. Detecting rhythms in time series with RAIN. *Journal of Biological Rhythms* **29,** 391–400 (2014).

2. Ruf, T. The Lomb-Scargle Periodogram in Biological Rhythm Research: Analysis of Incomplete and Unequally Spaced Time-Series. *Biological Rhythm Research* **30,** 178–201 (1999).

3. Talmy, D. *et al.* Contrasting controls on microzooplankton grazing and viral infection of microbial prey. *Frontiers in Marine Science* **6,** 182 (2019).

4. Berg, H. C. & Purcell, E. M. Physics of chemoreception. *Biophysical Journal* **20,** 193–219 (1977).

5. Murray, A. G. & Jackson, G. A. Viral dynamics: a model of the effects of size, shape, motion and abundance of single-celled planktonic organisms and other particles. *Marine Ecology Progress Series,* 103–116 (1992).

6. Von Smoluchowski, M. Investigation of a mathematical theory on the coagulation of colloidal suspensions. *Z. Physik. Chem.(Ger.)* **92,** 155 (1917).

7. Murata, K. *et al.* Visualizing adsorption of cyanophage P-SSP7 onto marine *Prochlorococcus. Scientific Reports* **7,** 44176 (2017).

8. Einstein, A. Über die von der molekularkinetischen Theorie der Wärme geforderte Bewegung von in ruhenden Flüssigkeiten suspendierten Teilchen. *Annalen der physik* **322,** 549–560 (1905).

9. Karl, D. M. & Church, M. J. Microbial oceanography and the Hawaii Ocean Time-series programme. *Nature Reviews Microbiology* **12,** 699–713 (2014).

10. Biller, S. J., Berube, P. M., Lindell, D. & Chisholm, S. W. *Prochlorococcus*: the structure and function of collective diversity. *Nature Reviews Microbiology* **13,** 13 (2015).

11. Zehr, J. P., Weitz, J. S. & Joint, I. How microbes survive in the open ocean. *Science* **357,** 646–647 (2017).

12. Kiørboe, T. How zooplankton feed: mechanisms, traits and trade-offs. *Biological Reviews* **86,** 311–339 (2011).

13. Kiørboe, T. *A mechanistic approach to plankton ecology* (Princeton University Press, 2008).

14. Ribalet, F. *et al.* SeaFlow data v1, high-resolution abundance, size and biomass of small phytoplankton in the North Pacific. *Scientific Data* **6,** 1–8 (2019).

15. Menden-Deuer, S. & Lessard, E. J. Carbon to volume relationships for dinoflagellates, diatoms, and other protist plankton. *Limnology and Oceanography* **45,** 569–579 (2000).

16. Mruwat, N. *et al.* A single-cell polony method reveals low levels of infected *Prochlorococcus* in oligotrophic waters despite high cyanophage abundances. *The ISME Journal* **15,** 41–54 (2021).

17. Connell, P. E., Ribalet, F., Armbrust, E. V., White, A. E. & Caron, D. A. Diel oscillations in feeding strategies of heterotrophic and mixotrophic nanoplankton in the North Pacific Subtropical Gyre. *Aquatic Microbial Ecology* **85,** 167–181 (2020).

18. Verity, P. G., Stoecker, D. K., Sieracki, M. E. & Nelson, J. R. Grazing, growth and mortality of microzooplankton during the 1989 North Atlantic spring bloom at 47 N, 18 W. *Deep Sea Research Part I: Oceanographic Research Papers* **40,** 1793–1814 (1993).

19. Neuer, S. & Cowles, T. J. Protist herbivory in the Oregon upwelling system. *Marine ecology progress series.* **113,** 147–162 (1994).

20. Karayanni, H., Christaki, U., Van Wambeke, F., Thyssen, M. & Denis, M. Heterotrophic nanoflagellate and ciliate bacterivorous activity and growth in the northeast Atlantic Ocean: a seasonal mesoscale study. *Aquatic Microbial Ecology* **51,** 169–181 (2008).

21. Ribalet, F. *et al.* Light-driven synchrony of *Prochlorococcus* growth and mortality in the subtropical Pacific gyre. *Proceedings of the National Academy of Sciences* **112,** 8008–8012 (2015).

22. Binder, B. J. & DuRand, M. D. Diel cycles in surface waters of the equatorial Pacific. *Deep Sea Research Part II: Topical Studies in Oceanography* **49,** 2601–2617 (2002).

23. Berube, P. M., Rasmussen, A., Braakman, R., Stepanauskas, R. & Chisholm, S. W. Emergence of trait variability through the lens of nitrogen assimilation in *Prochlorococcus. eLife* **8,** e41043 (2019).

24. Talmy, D. *et al.* An empirical model of carbon transfer to marine viruses and zooplankton grazers. *Environmental Microbiology* **21,** 2171–2181 (2019).

25. Liu, R., Liu, Y., Chen, Y., Zhan, Y. & Zeng, Q. Cyanobacterial viruses exhibit diurnal rhythms during infection. *Proceedings of the National Academy of Sciences* **116,** 14077–14082 (2019).

26. Demory, D. *et al.* Linking light-dependent life history traits with population dynamics for *Prochlorococcus* and cyanophage. *mSystems* **5,** e00586–19 (2020).

27. Ge, H., Xu, K. & Ghahramani, Z. *Turing: a language for flexible probabilistic inference* in *International Conference on Artificial Intelligence and Statistics, AISTATS 2018, 9-11 April 2018, Playa Blanca, Lanzarote, Canary Islands, Spain* (2018), 1682–1690. http://proceedings.mlr.press/v84/ge18b.html.

28. Bezanson, J., Edelman, A., Karpinski, S. & Shah, V. B. Julia: A fresh approach to numerical computing. *SIAM review* **59,** 65–98. https://doi.org/10.1137/141000671 (2017).

29. Lambert, B. *A student's guide to Bayesian statistics* (Sage, 2018).

30. Hoffman, M. D. & Gelman, A. The No-U-Turn sampler: adaptively setting path lengths in Hamiltonian Monte Carlo. *J. Mach. Learn. Res.* **15,** 1593–1623 (2014).

31. Mattern, J. P. *et al.* A Bayesian approach to modeling phytoplankton population dynamics from size distribution time series. *PLoS Computational Biology* **18,** e1009733 (2022).

32. Link, W. A. & Eaton, M. J. On thinning of chains in MCMC. *Methods in ecology and evolution* **3,** 112–115 (2012).

33. Gelman, A., Carlin, J. B., Stern, H. S. & Rubin, D. B. *Bayesian Data Analysis* (Chapman and Hall/CRC, 1995).

34. Casey, J. R., Björkman, K. M., Ferrón, S. & Karl, D. M. Size dependence of metabolism within marine picoplankton populations. *Limnology and Oceanography* **64,** 1819–1827 (2019).

35. Straile, D. Gross growth efficiencies of protozoan and metazoan zooplankton and their dependence on food concentration, predator-prey weight ratio, and taxonomic group. *Limnology and Oceanography* **42,** 1375–1385 (1997).

36. Li, Q., Edwards, K. F., Schvarcz, C. R., Selph, K. E. & Steward, G. F. Plasticity in the grazing ecophysiology of *Florenciella* (Dichtyochophyceae), a mixotrophic nanoflagellate that consumes *Prochlorococcus* and other bacteria. *Limnology and Oceanography* **66,** 47–60 (2021).

37. Zubkov, M. V. & Tarran, G. A. High bacterivory by the smallest phytoplankton in the North Atlantic Ocean. *Nature* **455,** 224–226 (2008).

38. Li, Q., Edwards, K. F., Schvarcz, C. R. & Steward, G. F. Broad phylogenetic and functional diversity among mixotrophic consumers of *Prochlorococcus*. *The ISME journal* **16,** 1557–1569 (2022).

39. Cai, L. *et al.* Abundant and cosmopolitan lineage of cyanopodoviruses lacking a DNA polymerase gene. *The ISME Journal,* 1–11. ISSN: 1751-7362 (2022).

40. Richardson, T. L. & Jackson, G. A. Small phytoplankton and carbon export from the surface ocean. *Science* **315,** 838–840 (2007).

41. Guidi, L. *et al.* Plankton networks driving carbon export in the oligotrophic ocean. *Nature* **532,** 465 (2016).

42. Richardson, T. L. Mechanisms and pathways of small-phytoplankton export from the surface ocean. *Annual Review of Marine Science* **11,** 57–74 (2019).

43. Sullivan, M. B., Weitz, J. S. & Wilhelm, S. Viral ecology comes of age. *Environmental Microbiology Reports* **9,** 33–35 (2017).

44. Zhao, Z. *et al.* Microbial transformation of virus-induced dissolved organic matter from picocyanobacteria: coupling of bacterial diversity and DOM chemodiversity. *The ISME Journal* **13,** 2551–2565 (2019).

45. Xiao, X. *et al.* Viral lysis alters the optical properties and biological availability of dissolved organic matter derived from picocyanobacteria *Prochlorococcus*. *Applied and Environmental Microbiology* **87,** e02271–20. ISSN: 0099-2240. eprint: https://aem.asm.org/content/early/2020/11/09/AEM.02271-20.full.pdf. https://aem.asm.org/content/early/2020/11/09/AEM.02271-20 (2021).

46. Zheng, Q. *et al.* Highly enriched N-containing organic molecules of *Synechococcus* lysates and their rapid transformation by heterotrophic bacteria. *Limnology and Oceanography* **66,** 335–348 (2021).

47. Iuculano, F., Mazuecos, I. P., Reche, I. & Agustí, S. *Prochlorococcus* as a possible source for transparent exopolymer particles (TEP). *Frontiers in Microbiology* **8,** 709 (2017).

48. Cruz, B. N. & Neuer, S. Heterotrophic bacteria enhance the aggregation of the marine picocyanobacteria *Prochlorococcus* and *Synechococcus*. *Frontiers in Microbiology* **10,** 1864 (2019).

49. Baines, S. B. *et al.* Significant silicon accumulation by marine picocyanobacteria. *Nature Geoscience* **5,** 886–891 (2012).

50. Krause, J. W. *et al.* Picoplankton contribution to biogenic silica stocks and production rates in the Sargasso Sea. *Global Biogeochemical Cycles* **31,** 762–774 (2017).

51. Wei, Y. *et al.* Significant contribution of picoplankton size fraction to biogenic silica standing stocks in the Western Pacific Ocean. *Progress in Oceanography* **192,** 102516 (2021).

52. Llabrés, M., Agustí, S. & Herndl, G. J. Diel in situ picophytoplankton cell death cycles coupled with cell division. *Journal of Phycology* **47,** 1247–1257 (2011).

53. Mella-Flores, D. *et al. Prochlorococcus* and *Synechococcus* have evolved different adaptive mechanisms to cope with light and UV stress. *Frontiers in Microbiology* **3,** 285 (2012).

54. Stramska, M. & Dickey, T. D. Short-term variability of the underwater light field in the oligotrophic ocean in response to surface waves and clouds. *Deep Sea Research Part I: Oceanographic Research Papers* **45,** 1393–1410 (1998).

55. Demory, D. *et al.* How do microalgae perceive light in a high-rate pond? Towards more realistic Lagrangian experiments. *Royal Society Open Science* **5,** 180523 (2018).

56. Kulk, G., van de Poll, W. H., Visser, R. J. & Buma, A. G. Low nutrient availability reduces high-irradiance-induced viability loss in oceanic phytoplankton. *Limnology and Oceanography* **58,** 1747–1760 (2013).

57. Mann, E. L. & Chisholm, S. W. Iron limits the cell division rate of *Prochlorococcus* in the eastern equatorial Pacific. *Limnology and Oceanography* **45,** 1067–1076 (2000).

58. Mann, E. L., Ahlgren, N., Moffett, J. W. & Chisholm, S. W. Copper toxicity and cyanobacteria ecology in the Sargasso Sea. *Limnology and Oceanography* **47,** 976–988 (2002).

59. Sarker, I., Moore, L. R. & Tetu, S. G. Investigating zinc toxicity responses in marine *Prochlorococcus* and *Synechococcus. Microbiology* **167,** 001064 (2021).

60. Pittera, J. *et al.* Connecting thermal physiology and latitudinal niche partitioning in marine *Synechococcus. The ISME journal* **8,** 1221–1236 (2014).

61. Baker, K. G. & Geider, R. J. Phytoplankton mortality in a changing thermal seascape. *Global Change Biology* **27,** 5253–5261 (2021).

62. Vardi, A. *et al.* A stress surveillance system based on calcium and nitric oxide in marine diatoms. *PLOS Biology* **4,** e60 (2006).

63. Bidle, K. D. The molecular ecophysiology of programmed cell death in marine phytoplankton. *Annual Review of Marine Science* **7,** 341–375 (2015).

64. Bidle, K. D. Programmed cell death in unicellular phytoplankton. *Current Biology* **26,** R594–R607 (2016).

65. Morris, J. J., Kirkegaard, R., Szul, M. J., Johnson, Z. I. & Zinser, E. R. Facilitation of robust growth of *Prochlorococcus* colonies and dilute liquid cultures by "helper" heterotrophic bacteria. *Applied and Environmental Microbiology* **74,** 4530–4534 (2008).

66. Morris, J. J., Johnson, Z. I., Szul, M. J., Keller, M. & Zinser, E. R. Dependence of the cyanobacterium *Prochlorococcus* on hydrogen peroxide scavenging microbes for growth at the ocean's surface. *PLOS ONE* **6,** e16805 (2011).

67. Fucich, D. & Chen, F. Presence of toxin-antitoxin systems in picocyanobacteria and their ecological implications. *The ISME Journal* **14,** 2843–2850 (2020).

68. Moger-Reischer, R. Z. & Lennon, J. T. Microbial ageing and longevity. *Nature Reviews Microbiology* **17,** 679–690 (2019).

69. Franklin, D. J. Explaining the causes of cell death in cyanobacteria: what role for asymmetric division? *Journal of Plankton Research* **36,** 11–17 (2013).

70. Roth-Rosenberg, D. *et al. Prochlorococcus* Cells Rely on Microbial Interactions Rather than on Chlorotic Resting Stages To Survive Long-Term Nutrient Starvation. *mBio* **11,** e01846–20 (2020).

71. Kearney, S. M., Thomas, E., Coe, A. & Chisholm, S. W. Microbial Diversity of Co-occurring Heterotrophs in Cultures of Marine Picocyanobacteria. *Environmental Microbiome* **16,** 1–15 (1 2021).

72. Moore, L. R. & Chisholm, S. W. Photophysiology of the marine cyanobacterium *Prochlorococcus*: ecotypic differences among cultured isolates. *Limnology and Oceanography* **44,** 628–638 (1999).

73. Martiny, A. C., Coleman, M. L. & Chisholm, S. W. Phosphate acquisition genes in *Prochlorococcus* ecotypes: evidence for genome-wide adaptation. *Proceedings of the National Academy of Sciences* **103,** 12552–12557 (2006).

74. Tolonen, A. C. *et al.* Global gene expression of *Prochlorococcus* ecotypes in response to changes in nitrogen availability. *Molecular systems biology* **2,** 53 (2006).

75. Johnson, Z. I. *et al.* Niche partitioning among *Prochlorococcus* ecotypes along ocean-scale environmental gradients. *Science* **311,** 1737–1740 (2006).

76. Zinser, E. R. *et al.* Influence of light and temperature on *Prochlorococcus* ecotype distributions in the Atlantic Ocean. *Limnology and Oceanography* **52,** 2205–2220 (2007).

77. Thompson, A. W., Huang, K., Saito, M. A. & Chisholm, S. W. Transcriptome response of high-and low-light-adapted *Prochlorococcus* strains to changing iron availability. *The ISME Journal* **5,** 1580–1594 (2011).
