# Supplementary material for: Disentangling top-down drivers of mortality underlying diel population dynamics of Prochlorococcus in the North Pacific Subtropical Gyre
Source: Nat Commun. 2024 Mar 7;15:2105. doi: 10.1038/s41467-024-46165-3 (PMC10920773; doi:10.1038/s41467-024-46165-3)
Supplement: Supplementary file 3 — Reporting Summary [file 41467_2024_46165_MOESM3_ESM.pdf]

nature portfolio

Corresponding author(s): Stephen Beckett, David Demory, Joshua Weitz

Last updated by author(s): Jan 17, 2024

Reporting Summary

Nature Portfolio wishes to improve the reproducibility of the work that we publish. This form provides structure for consistency and transparency in reporting. For further information on Nature Portfolio policies, see our Editorial Policies and the Editorial Policy Checklist.

Statistics

For all statistical analyses, confirm that the following items are present in the figure legend, table legend, main text, or Methods section.

n/a	Confirmed	
☐	☒	The exact sample size (n) for each experimental group/condition, given as a discrete number and unit of measurement
☐	☒	A statement on whether measurements were taken from distinct samples or whether the same sample was measured repeatedly
☐	☒	The statistical test(s) used AND whether they are one- or two-sided<br>*Only common tests should be described solely by name; describe more complex techniques in the Methods section.*
☒	☐	A description of all covariates tested
☐	☒	A description of any assumptions or corrections, such as tests of normality and adjustment for multiple comparisons
☐	☒	A full description of the statistical parameters including central tendency (e.g. means) or other basic estimates (e.g. regression coefficient) AND variation (e.g. standard deviation) or associated estimates of uncertainty (e.g. confidence intervals)
☒	☐	For null hypothesis testing, the test statistic (e.g. F, t, r) with confidence intervals, effect sizes, degrees of freedom and P value noted<br>*Give P values as exact values whenever suitable.*
☐	☒	For Bayesian analysis, information on the choice of priors and Markov chain Monte Carlo settings
☒	☐	For hierarchical and complex designs, identification of the appropriate level for tests and full reporting of outcomes
☒	☐	Estimates of effect sizes (e.g. Cohen's d, Pearson's r), indicating how they were calculated

Our web collection on statistics for biologists contains articles on many of the points above.

Software and code

Policy information about availability of computer code

Data collection	Data collection was performed prior to this manuscript. For specifics we refer readers to Ribelet et al., 2020, doi: 10.5281/zenodo.3994953 for Prochlorococcus cell abundance; Mruwat et al., 2021, doi: 10.1038/s41396-020-00752-6 for virus abundance and infection; and Connell et al. 2020, doi: 10.3354/ame01950 for measurements of heterotrophic nanoflagellates.
Data analysis	All code used for data analysis and figure plotting is publicly archived in Zenodo (https://zenodo.org/doi/10.5281/zenodo.4917226). Software used in our analyses and plotting are: MATLAB 9.12.0.1927505 (R2022a) Update 1, and the Econometrics ToolBox; Julia v 1.6.3 and packages CSV v0.10.3, DataFrames v1.3.2, Distributions v0.25.49, Measures v0.3.1, LaTeXStrings v1.3.0, CairoMakie v0.7.4., MCMCChains v5.1.0, Plots v1.27.0, StatsPlots v0.14.33, DifferentialEquations v7.1.0, Turing v0.20.4, MCMCChains v5.1.0; R version 4.2.1 (2022-06-23) and packages rnaturalearth 0.1.0, vroom 1.5.7, sf 1.0-8, lubridate 1.8.0, lomb 2.1.0, RAIN 1.34.0 (from Bioconductor).

For manuscripts utilizing custom algorithms or software that are central to the research but not yet described in published literature, software must be made available to editors and reviewers. We strongly encourage code deposition in a community repository (e.g. GitHub). See the Nature Portfolio guidelines for submitting code & software for further information.

Data

Policy information about availability of data

All manuscripts must include a data availability statement. This statement should provide the following information, where applicable:

- Accession codes, unique identifiers, or web links for publicly available datasets
- A description of any restrictions on data availability
- For clinical datasets or third party data, please ensure that the statement adheres to our policy

The data sources that we use in our analysis have been published. Prochlorococcus cell abundances are from Ribalet et al. 2020, doi: 10.5281/zenodo.3994953 , free virus and percentage of infected Prochlorococcus cells are from Mruwat et al. 2021, doi: 10.1038/s41396-020-00752-6, and heterotrophic nanoflagellate abundances are from Connell et al. 2020, doi: 10.3354/ame01950. All data from analyses, including for ECLIP and Bayesian parameter inference are archived on Zenodo, doi: 10.5281/zenodo.10127185.

Research involving human participants, their data, or biological material

Policy information about studies with human participants or human data. See also policy information about sex, gender (identity/presentation), and sexual orientation and race, ethnicity and racism.

Reporting on sex and gender	N/A
Reporting on race, ethnicity, or other socially relevant groupings	N/A
Population characteristics	N/A
Recruitment	N/A
Ethics oversight	N/A

Note that full information on the approval of the study protocol must also be provided in the manuscript.

Field-specific reporting

Please select the one below that is the best fit for your research. If you are not sure, read the appropriate sections before making your selection.

☐ Life sciences ☐ Behavioural & social sciences ☒ Ecological, evolutionary & environmental sciences

For a reference copy of the document with all sections, see nature.com/documents/nr-reporting-summary-flat.pdf

Ecological, evolutionary & environmental sciences study design

All studies must disclose on these points even when the disclosure is negative.

Study description	This study aimed to monitor microbial ecological activity over the diel cycle via repeated sampling of surface waters (15m) on a Lagrangian cruise track over the course of several days in the North Pacific Subtropical Gyre.
Research sample	This study aimed to monitor microbial ecological activity over the diel cycle via repeated sampling of surface waters (15m) on a Lagrangian cruise track over the course of several days in the North Pacific Subtropical Gyre.
Sampling strategy	For specifics on data collection please refer to Ribelet et al., 2020, doi: 10.5281/zenodo.3994953 for Prochlorococcus cell abundance; Mruwat et al., 2021, doi: 10.1038/s41396-020-00752-6 for virus abundance and infection; and Connell et al. 2020, doi: 10.3354/ame01950 for measurements of heterotrophic nanoflagellates.
Data collection	For specifics on data collection please refer to Ribelet et al., 2020, doi: 10.5281/zenodo.3994953 for Prochlorococcus cell abundance; Mruwat et al., 2021, doi: 10.1038/s41396-020-00752-6 for virus abundance and infection; and Connell et al. 2020, doi: 10.3354/ame01950 for measurements of heterotrophic nanoflagellates.
Timing and spatial scale	Data were collected between 24 July 2015 and 4 August 2015 across an anticyclonic eddy in the North Pacific. Flow cytometry data were collected roughly every 3 minutes, with samples for cyanophage, infected cells and heterotrophic nanoflagellate grazers being made every 4 hours over two periods as highlighted in Fig1c. For specifics on data collection please refer to Ribelet et al., 2020, doi: 10.5281/zenodo.3994953 for Prochlorococcus cell abundance; Mruwat et al., 2021, doi: 10.1038/s41396-020-00752-6 for virus abundance and infection; and Connell et al. 2020, doi: 10.3354/ame01950 for measurements of heterotrophic nanoflagellates.
Data exclusions	No data were excluded from model-data fitting.

Reproducibility	Data collected were observational. We developed code to analyze this data in a reproducible manner. In particular, for our model-data inference we checked for reproducibility by using multiple chains in our Bayesian inference scheme and assessed their convergence.
Randomization	Data collected were observational and data randomization was not relevant to this study.
Blinding	Collected data were observational and blinding was not relevant.

Did the study involve field work? ☒ Yes ☐ No

Field work, collection and transport

Field conditions	A full description of the environmental conditions are available at CMAP: https://simonscmap.dev/catalog/datasets/KM1513_HOE_Legacy_2A
Location	Fieldwork was conducted between 25 July to 5 August, 2015 in the oligotrophic North Pacific Subtropical Gyre. To maximize the signal to noise ratio in an open ocean environment, a Lagrangian sampling strategy was implemented whereby World Ocean Circulation Experiment Surface Velocity Profile (WOCE SVP) drifters from Pacific Gyre, Inc. were deployed within the center of a mesoscale anticyclonic eddy. The mesoscale eddy fields were identified using Archiving, Validation, and Interpretation of Satellite Oceanographic data (AVISO) and when the field sampling occurred, the target anticyclonic eddy was located north of the Hawaiian Islands at 24.2 N and 156.5 W, with a diameter of ~100km. Over the 12-day sampling period, the shipboard measurements were conducted alongside the drifters as they performed an almost complete circular pattern with a diameter of ~44km (Figure 1c). SeaFlow measurements were taken from the underway. Water-column seawater sampling for diel measurements took place every 4 h for a period of 4 days (26-30 July) and 3 days (31 July - 3 August) at a depth of 15 m corresponding to the depth of the drogue.
Access & import/export	For specifics on data collection please refer to Ribelet et al., 2020, doi: 10.5281/zenodo.3994953 for Prochlorococcus cell abundance; Mruwat et al., 2021, doi: 10.1038/s41396-020-00752-6 for virus abundance and infection; and Connell et al. 2020, doi: 10.3354/ame01950 for measurements of heterotrophic nanoflagellates.
Disturbance	Samples were collected using standard oceanographic methods with minimum disturbance to the surrounding waters.

Reporting for specific materials, systems and methods

We require information from authors about some types of materials, experimental systems and methods used in many studies. Here, indicate whether each material, system or method listed is relevant to your study. If you are not sure if a list item applies to your research, read the appropriate section before selecting a response.

Materials & experimental systems

n/a	Involved in the study	
☒	☐	Antibodies
☒	☐	Eukaryotic cell lines
☒	☐	Palaeontology and archaeology
☒	☐	Animals and other organisms
☒	☐	Clinical data
☒	☐	Dual use research of concern
☒	☐	Plants

Methods

n/a	Involved in the study	
☒	☐	ChIP-seq
☒	☐	Flow cytometry
☒	☐	MRI-based neuroimaging

Plants

Seed stocks	N/A
Novel plant genotypes	N/A
Authentication	N/A
